# Case Report: Suspected low-pressure hydrocephalus following surgical treatment of traumatic brain injury in a patient with subsequently diagnosed glioblastoma

**DOI:** 10.3389/fonc.2026.1721506

**Published:** 2026-06-29

**Authors:** Shuanghong He, Lirui Dai, Wenyi Zhan, Shu Jiang, Peizhi Zhou

**Affiliations:** 1 Health Management Center, West China Hospital of Sichuan University, Sichuan University, Chengdu, Sichuan, China; 2 Department of Neurosurgery, West China Hospital of Sichuan University, Sichuan University, Chengdu, Sichuan, China

**Keywords:** bone marrow suppression, glioblastoma, low-pressure hydrocephalus, surgery, traumatic brain injury

## Abstract

Glioblastoma (GBM) represents the most prevalent primary malignant neoplasm of the central nervous system, frequently presenting with clinical manifestations such as elevated intracranial pressure, focal neurological deficits, and seizures. The incidental detection and diagnosis of glioblastoma two months subsequent to surgical intervention for traumatic brain injury (TBI) is exceptionally uncommon, and a causal link between TBI and subsequent glioma development remains unsubstantiated. To our knowledge, reports of low intracranial pressure hydrocephalus as a consequence of tumor resection surgery are very limited. In this report, we detail the case of a 50-year-old female patient who underwent surgical management for TBI. Two months postoperatively, space-occupying lesions were identified, prompting surgical resection, which confirmed the diagnosis of GBM through pathological examination. Following surgery, the patient received standard radiotherapy and chemotherapy. During her treatment course, she was hospitalized due to complications related to bone marrow suppression and suspected low-pressure hydrocephalus. Despite exhaustive therapeutic interventions, the patient ultimately succumbed to diffuse cerebral edema and brain herniation. This rare case involves a patient who, two months post-surgery for traumatic brain injury, unexpectedly identified a space-occupying lesion in the brain. Subsequent diagnostic evaluation confirmed the lesion as glioblastoma. Despite comprehensive treatment, the patient ultimately succumbed to tumor recurrence, bone marrow suppression, and low intracranial pressure. This case serves as a reference for the incidental discovery and subsequent treatment strategies in asymptomatic GBM patients, while also providing insights into treatment and research approaches for suspected low-pressure hydrocephalus occurring during the disease course. While noting that only a temporal association—not causation—can be inferred between TBI and GBM based on this single report.

## Introduction

Glioblastoma (GBM) is the most prevalent primary malignant neoplasm of the central nervous system, characterized by its high invasiveness, rapid progression, and notably poor prognosis (1). Approximately 30% of patients initially present with epilepsy, later developing clinical manifestations such as increased intracranial pressure and focal neurological deficits (2). Treatment strategies include maximal safe surgical resection of the tumor, concurrent chemoradiotherapy, and targeted or immunotherapy approaches. Under standard treatment protocols, the median survival for patients with IDH wild-type GBM is approximately 14–16 months, whereas for those with the IDH mutant type, survival can extend to 3–5 years (3–5).

The occurrence of GBM subsequent to traumatic brain injury (TBI) is exceedingly rare, and its potential role as a pathogenic factor in GBM development remains a topic of ongoing debate. A limited number of prior studies have documented the emergence of various grades of gliomas in patients within 5 to 35 years post-TBI, with high-grade gliomas being relatively uncommon (6–11). In the current study, a patient who underwent surgical intervention for TBI, without any preceding symptoms, was subsequently diagnosed with GBM and received a comprehensive treatment regimen. It is pertinent to consider whether the progression of the patient’s condition is related to the recent cranial trauma surgery and whether this surgical intervention is associated with the patient’s disease progression and prognosis.

To date, there have been no documented instances of GBM being identified subsequent to TBI, nor of the emergence of suspected low-pressure hydrocephalus following treatment. Two months postoperatively, a space-occupying lesion was detected in the patient’s brain, necessitating surgical resection. Following the resection, the patient underwent standard radiotherapy and chemotherapy. Four months after the surgery, the patient developed low intracranial pressure hydrocephalus. Eight months subsequent to the initial operation, the patient was admitted to our hospital in critical condition due to sudden cardiac arrest. Despite the implementation of lateral ventricular drilling and drainage, the patient ultimately succumbed to complications associated with suspected low-pressure hydrocephalus, ischemia, and hypoxia of brain tissue, which resulted in diffuse brain swelling and brain herniation. Whether this temporal association reflects causality, accelerated growth of a pre-existing lesion, or mere coincidence remains unknown.

## Case illustration

### Initial presentation

A 50-year-old Chinese female patient suffered from acute epidural hematoma on the left side due to trauma and received surgical treatment at the local hospital immediately (The patient’s family members were unable to provide the imaging data from the local hospital). Two months after the surgery, an unexpected space-occupying lesion was found at the tip of the left temporal lobe of the patient. The patient had not typically experienced symptoms such as dizziness, headache, epilepsy, or other discomforts. To further investigate the nature of the lesion and facilitate its surgical excision, the patient was admitted to the Department of Neurosurgery for additional treatment. Upon admission, the patient underwent multimodal magnetic resonance imaging (MRI) of the brain tumor. The imaging revealed an irregular mass in the left frontotemporal lobe, measuring approximately 3.55 × 5.2 cm (**Figures 1A–C**). The mass exhibited uneven enhancement, accompanied by edema of the surrounding brain parenchyma and significantly increased perfusion (**Figure 1D**). However, no significant elevation was observed in the tumor spectrum (**Figure 1E**), leaving the precise nature of the tumor indeterminate at this stage. Based on clinical experience, the possibility of glioma or meningioma could not be excluded. Differential diagnoses at this stage included high-grade glioma, primary central nervous system lymphoma, metastatic disease, and atypical meningioma. The absence of systemic malignancy on whole-body imaging, the characteristic irregular enhancement with central necrosis on MRI, and elevated relative cerebral blood volume on perfusion imaging favored a high-grade glial neoplasm over lymphoma or metastasis. Nevertheless, definitive diagnosis required histopathological confirmation.

**Figure 1:**
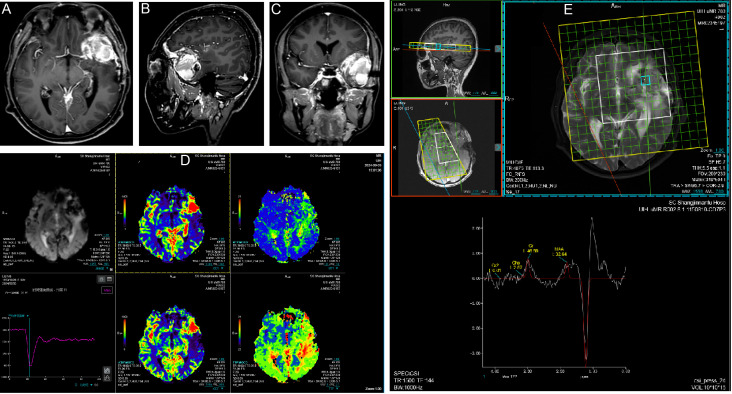
Multimodal MRI examination of brain tumors: **(A–C)** irregular masses can be seen in the left frontotemporal lobe; **(D)** perfusion imaging scan of brain tissue; **(E)** tumor spectrum.

### Preoperative risk analysis and surgical plan

Our department possesses substantial clinical expertise in the resection of space-occupying lesions within this anatomical region. The primary risks associated with this surgical intervention include: (1) significant intraoperative hemorrhage and postoperative rebleeding; (2) postoperative neurological deficits; (3) postoperative hydrocephalus; (4) postoperative infection and cerebrospinal fluid leakage; (5) incomplete resection of the lesion, which may result in recurrence contingent upon the lesion’s pathological characteristics; and (6) other associated risks. Following comprehensive discussions with the patient and their family regarding the treatment plan, a decision was made to proceed with a craniotomy to excise the affected tissue.

### Surgical procedure

Under general anesthesia, the patient was positioned in a supine orientation, and the incision was extended from the initial temporal craniotomy to facilitate a left frontotemporal approach for surgical access. The original bone window was removed and enlarged to provide enhanced access, extending anteriorly to the anterior portion of the middle cranial fossa, posteriorly to the anterior segment of the external auditory canal, superiorly to the inferior frontal gyrus, and inferiorly to the middle base of the skull. This expansion enabled exposure of the collateral fissure vessels. During the procedure, significant thickening of the dura mater and extensive pathological vascular formations were observed. Upon incision of the dura mater, elevated intracranial pressure was detected, with the brain tissue appearing protruded. Abnormal lesions adhered to the dura mater were visible on the brain surface, characterized by a fish-flesh appearance and invasive growth in multiple directions. Some lesions exhibited indistinct boundaries with normal brain tissue and contained areas of cystic necrosis. Tissue samples were excised for pathological examination. Employing electrophysiological monitoring, the lesion was meticulously resected, along with the involved and invaded dura mater. The total resected lesion measured approximately 4.5 × 4.5 cm. After thorough hemostasis, repair the dura mater to prevent cerebrospinal fluid leakage, and finally close the skull layer by layer.

### Postoperative management

Following the surgical procedure, the patient underwent a regimen of treatments that included dehydration management, gastric protection, prophylaxis against epilepsy and infection, administration of antiemetics, blood volume supplementation, and nutritional support. On the first postoperative day, computed tomography (CT) imaging revealed the resection of the tumor, as compared to preoperative images (**Supplementary Figure 1A**). Additionally, a minor presence of effusion, gas, and blood accumulation was observed in the surgical site (**Supplementary Figure 1B**). After a week of targeted symptomatic treatment, the patient was discharged with stable vital signs and intracranial conditions. At the time of discharge, the patient exhibited no symptoms such as headache, nausea, vomiting, altered consciousness, or motor function impairment.

### Pathological biopsy and postoperative treatment

The lesion in the temporal lobe was selected for further analysis, and the pathological diagnosis confirmed glioblastoma (WHO grade 4). Immunohistochemical analysis demonstrated that the tumor cells were positive for GFAP, Oligo2, and partially positive for EMA and PR, while negative for CK(Pan). Molecular pathology results indicated an IDH wild type, absence of MGMT methylation, a mutation at position 228 (C>T) in the TERT gene promoter region, and no mutation detected at position 250 (**Supplementary Figure 2**). Following the surgical procedure, the patient underwent a regimen of regular radiotherapy and temozolomide chemotherapy. Postoperatively, the patient received 30 sessions of radiotherapy. During this period, temozolomide chemotherapy was administered concurrently, at a dose of 75 mg/square meter, once daily, for a total of 42 days. After the radiotherapy, chemotherapy was suspended for 28 days, and then an adjuvant chemotherapy phase began, with the dosage: 150–200 mg/square meter, once daily, for 5/28 days.

#### Follow-up: 4 months after the operation

A follow-up examination was conducted at our hospital four months post-operation. During this period, the patient reported no significant symptoms, such as headache, nausea, vomiting, or disturbances in consciousness. Furthermore, the neurological examination yielded no positive findings. However, a head MRI revealed ventricular system dilation, hydrocephalus formation, and a minor recurrence of the tumor in the temporal pole (**Figure 2**). The patient was advised to continue with chemotherapy.

**Figure 2:**
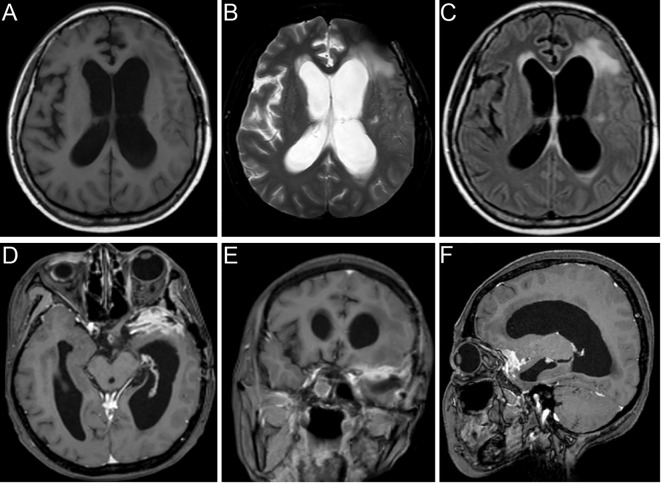
The enhanced MRI of the head was reexamined 4 months after the operation, and hydrocephalus was observed (ventricular enlargement, effacement of cortical sulci, periventricular lucency). **(A–D)** Axial position, **(E)** Coronal position, **(F)** Sagittal position.

#### Follow-up: 6 months after the operation - bone marrow suppression

Six months following the surgical procedure, the patient returned to our hospital for a follow-up examination, presenting with symptoms of skin ecchymosis and dark red urine. Laboratory analyses revealed a significant decline in platelet, white blood cell, and red blood cell counts, as well as reduced hemoglobin levels (Only routine blood tests were conducted, without performing more comprehensive hematological examinations). As a result, the patient was referred to the hematology department for symptomatic management. During the hospitalization period, the patient underwent transfusions of platelet and red blood cell suspensions. Simultaneously, therapeutic interventions included the administration of Juheli (human interleukin-11 injection), Tebiao (recombinant human thrombopoietin injection), Yibiao (human erythropoietin injection), and Jisaixin (human granulocyte-stimulating factor injection). After approximately 10 days of continuous treatment and monitoring, there was a notable increase in the levels of each cell line (**Figure 3**). A cranial CT scan indicated a slight recurrence of the tumor, with no significant change compared to 4 months after the operation, and there was no exacerbation of hydrocephalus compared to previous evaluations. However, a head MRI examination was not conducted (**Figures 4A–D**; **Supplementary Figure 3**). The severity of bone marrow suppression was not formally graded using a standard framework (e.g., CTCAE), and no additional hematologic evaluation (e.g., bone marrow biopsy, peripheral smear) was performed, as the clinical picture was consistent with chemotherapy/radiotherapy-induced myelosuppression and the patient improved with supportive care.

**Figure 3:**
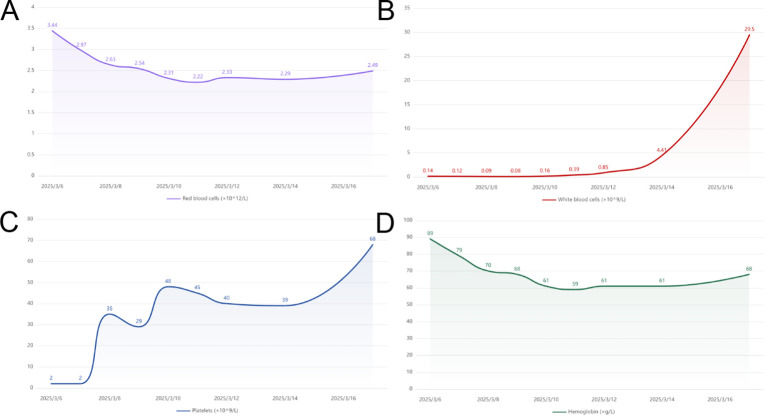
Six months after the operation, the patient experienced significant reduction in platelets, white blood cells and red blood cells due to bone marrow suppression after radiotherapy, and the hemoglobin level decreased. The changes of various indicators after symptomatic treatment. **(A)** Red blood cells, **(B)** White blood cells, **(C)** Platelets, **(D)** Hemoglobin.

**Figure 4:**
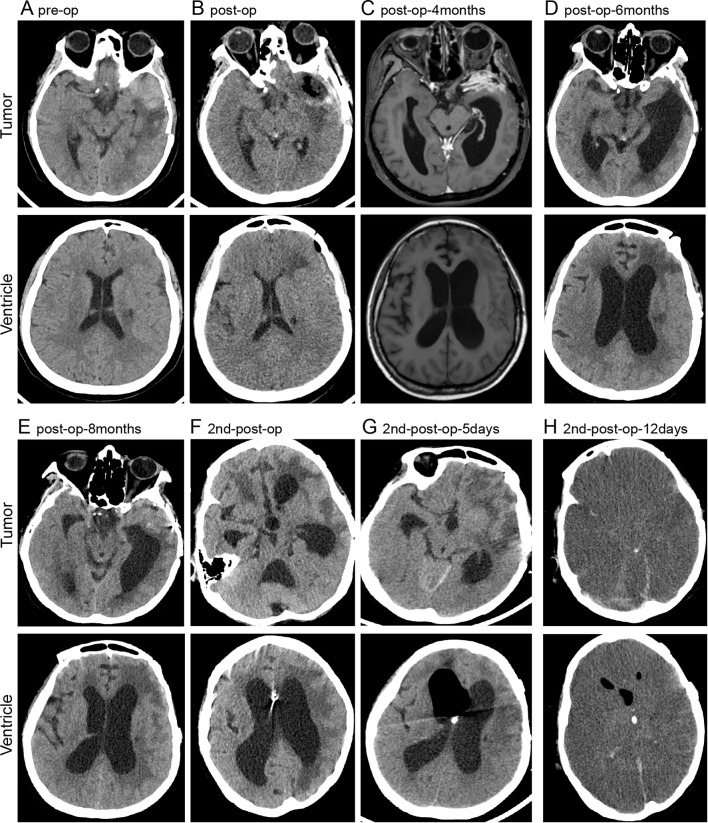
Graphs of changes in the intracranial condition of the patient at different time periods (including tumor recurrence and neurologic deterioration), including: **(A)** before the operation; **(B)** postoperative; **(C)** 4 months after the operation; **(D)** 6 months after the operation; **(E)** 8 months after the operation; **(F)** After the second operation; **(G)** 5 days after the second operation; **(H)** 12 days after the second operation.

#### Follow-up: 8 months after the operation - cardiac arrest

Eight months post-surgery, the patient presented to our hospital for follow-up and re-evaluation. The patient exhibited acute respiratory distress due to an excessive accumulation of phlegm, which was difficult to expel, leading to cardiac arrest. The emergency department team promptly initiated cardiopulmonary resuscitation and performed tracheal intubation. Approximately three minutes later, the patient’s spontaneous cardiac rhythm was restored. During this episode, there were no observed limb convulsions, ocular fixation, or oral frothing. A cranial CT scan revealed a significant recurrence of the tumor with potential hemorrhagic involvement. Additionally, hydrocephalus had worsened, and there was marked compression of the lateral ventricles compared to imaging conducted two months prior (**Figure 4E**). The patient was subsequently transferred to the intensive care unit (ICU) for further management.

#### Follow-up: 2nd operation - low pressure hydrocephalus

In light of the potential for cerebral edema resulting from hypoxia following cardiopulmonary resuscitation, in conjunction with the aggravation of hydrocephalus, a thorough discussion regarding the treatment plan was held with the patient’s family. Consequently, a left lateral ventricle drilling and drainage procedure was performed on the patient the same day (**Figure 4F**). Notably, despite the presence of severe hydrocephalus, the initial cerebrospinal fluid (CSF) drainage was sluggish. Upon the patient’s return to the intensive care unit post-operatively, the CSF drainage was completely obstructed. However, CSF was successfully aspirated from the drainage tube using a 20-milliliter syringe. As a result, approximately 100 milliliters of CSF have been manually extracted at the patient’s bedside daily since the procedure.

#### Follow-up: 12 days after 2nd operation - diffuse brain swelling

On the fifth postoperative day, a follow-up brain CT scan of the patient revealed hemorrhage in the left cerebellar hemisphere and the presence of scattered air in both ventricles (**Figure 4G**). By the tenth postoperative day, the patient exhibited anisocoria, with unequal pupil diameters, and a sluggish pupillary light reflex. On the twelfth postoperative day, the patient’s pupils were dilated, and the light reflex was absent. The brain CT indicated diffuse cerebral edema, with significant compression of the brainstem and ventricular system, rendering them nearly indistinguishable (**Figure 4H**). The Intensive Care Unit (ICU) continued to administer symptomatic and supportive care. However, on the fifteenth postoperative day, the patient’s family elected to discontinue treatment and voluntarily discharged the patient from the hospital. **Table 1** is the structured timeline.

**Table 1:** Clinical timeline.

Time point	Event	Key findings/interventions
Day 0	Left acute epidural hematoma	Emergency surgery at local hospital
Month 2	Incidental lesion on follow-up imaging	Asymptomatic; 3.55 × 5.2 cm mass on MRI
Month 2 + 1 week	GBM resection	Left frontotemporal craniotomy; gross total resection
Month 2 + 2 weeks	Pathological diagnosis	IDH wildtype, MGMT unmethylated, TERT mutation
Month 2–4	Adjuvant therapy	30 fractions RT (60 Gy) + concomitant TMZ
Month 4	First hydrocephalus	MRI: ventriculomegaly; minor tumor recurrence
Month 6	Bone marrow suppression	Platelets/WBC/RBC/Hb ↓; transfusions + growth factors
Month 8	Cardiac arrest	Post-CPR hypoxic brain injury
Month 8 + same day	LPH diagnosis	EVD placed; poor spontaneous drainage; daily aspiration
Month 8 + day 12	Diffuse brain swelling	Pupils fixed/dilated; CT: diffuse edema
Month 8 + day 15	Death	Family withdrew care

### Diagnostic assessment

A comprehensive multimodal brain MRI was conducted, incorporating T1-weighted, T2-weighted, FLAIR, contrast-enhanced T1, perfusion-weighted imaging (PWI), and MR spectroscopy sequences. The imaging identified an irregular mass in the left frontotemporal region, measuring 3.55 × 5.2 cm, characterized by heterogeneous enhancement, perilesional edema, and elevated relative cerebral blood volume (rCBV) on perfusion imaging, indicative of a high-grade glial neoplasm. MR spectroscopy did not reveal a significant elevation in the tumor spectrum, rendering it nondiagnostic. The differential diagnosis at this juncture included high-grade glioma (most probable), primary CNS lymphoma, atypical meningioma, and metastatic disease. Whole-body imaging showed no evidence of systemic malignancy, reducing the likelihood of metastasis.

Surgical specimens were processed with formalin fixation and paraffin embedding. Hematoxylin and eosin (H&E) staining revealed hypercellularity, nuclear atypia, mitotic figures, microvascular proliferation, and pseudopalisading necrosis, findings consistent with glioblastoma, WHO grade 4. Immunohistochemical analysis demonstrated positivity for GFAP and Olig2, supporting a glial origin, partial positivity for EMA and PR, and negativity for CK(Pan), thereby excluding carcinoma. Molecular testing revealed IDH wildtype, absence of MGMT promoter methylation, and a TERT promoter mutation (c.228C>T). No mutation was detected at position c.250. These molecular features are associated with primary glioblastoma and confer a poor prognosis, with a median overall survival of approximately 14–16 months under standard Stupp protocol therapy.

Several areas of diagnostic uncertainty remained throughout the disease course. First, the absence of pre-TBI brain imaging precluded determination of whether the GBM was pre-existing, developed *de novo* after trauma, or exhibited accelerated growth triggered by TBI or subsequent surgery. Second, the diagnosis of low-pressure hydrocephalus was inferred from clinical features (ventriculomegaly, poor spontaneous EVD drainage, ease of manual aspiration) without direct intracranial pressure measurement, as discussed in the Diagnostic limitations section. Third, the cause of bone marrow suppression was attributed to temozolomide and radiotherapy, but no bone marrow biopsy was performed to exclude other etiologies.

## Discussion

Glioblastoma (GBM) is the most common and fatal malignant tumor of the central nervous system (12). Modern treatment strategies have improved the overall prognosis. However, various therapeutic side effects often occur during the treatment process, such as a decline in function or cognitive ability. There are also many treatment challenges, such as how to safely remove the tumor to the greatest extent and then carry out adjuvant radiotherapy and temozolomide chemotherapy (13). Over the past few decades, the overall survival rate of patients with GBM has increased from 3.3 months to approximately 15 months (14). The incidence and mortality of traumatic brain injury (TBI) are both relatively high (15). The most important biological responses of TBI are neuroinflammation and oxidative stress (16). However, the incidence of GBM after TBI is extremely low, and most patients are diagnosed with glioma due to epilepsy or other clinical symptoms many years after TBI. In this instance, a brain lesion was identified in the intracranial region of the patient two months following surgery for TBI. It remains to be determined whether this lesion was pre-existing, whether it developed rapidly post-TBI, or whether it is part of a natural growth process.

Suleyman et al. once reported a 65-year-old patient who was admitted to the hospital due to right hemiplegia, epilepsy and consciousness disorder. Examination revealed an intracranial space-occupying lesion. Radical resection of the lesion tissue was performed, and the pathological diagnosis was GBM. Postoperative special radiotherapy and chemotherapy for the whole brain were carried out. No prognostic follow-up was conducted for the patients. Thirty-five years ago, this patient underwent surgical treatment for lacerations of the scalp and depressed fractures of the skull (7). Ranjith et al. once reported a case where a space-occupying lesion in the left frontal lobe was found due to increased intracranial pressure 5 years after TBI. Subsequently, the patient underwent radical resection of the mass, and the histopathological report was GBM. He completed whole brain extracorporeal radiotherapy and continued chemotherapy. Nine months after the operation, the patient died due to tumor recurrence (8). Han et al. proposed a case of post-traumatic GBM related to pregnancy. The patient at 16 weeks of gestation was admitted to the hospital due to left hemiplegia and epilepsy. Examination revealed a huge space-occupying lesion in the right frontal lobe, with severe edema around the lesion. Eventually, the patient terminated the pregnancy and underwent resection of the space-occupying lesion. The histopathology indicated GBM. The patient will undergo radiotherapy and chemotherapy after the operation. Long-term follow-up for this patient has not been conducted yet (9). Although epidemiological studies may not provide definitive conclusions, trauma could potentially act as an auxiliary carcinogen in the presence of initial carcinogenic factors. The cases reviewed herein revealed gliomas several years post-TBI, yet there is no conclusive evidence establishing a causal relationship between the two. Prior to the occurrence of TBI, the patients in this study exhibited no symptoms. However, within two months post-TBI surgery, intracranial space-occupying lesions were detected, accompanied by symptoms such as epilepsy. It is reasonable to hypothesize that lesions may have already been present in the patients’ intracranial regions at an early stage, and that the progression of these lesions was accelerated by the influence of TBI. However, the present case does not provide evidence for a causal relationship between TBI and subsequent GBM.

Low-pressure hydrocephalus (LPH) is a special type of hydrocephalus, characterized by enlarged ventricles but intracranial pressure (ICP) lower than the normal range (<5 cm H_2_O), and poor response to conventional cerebrospinal fluid shunt surgery. LPH is relatively rare, but it is difficult to identify clinically and is prone to misdiagnosis or delayed treatment. The exact pathogenesis of LPH remains unclear, but studies have shown that there are many risk factors, such as subarachnoid hemorrhage, craniocerebral radiotherapy, and cerebrospinal fluid leakage (17, 18). The main treatment methods include negative pressure drainage and third ventriculoplasty, and ventriculoperitoneal shunt when necessary. After ventricular drilling and drainage for this patient, cerebrospinal fluid drainage was difficult. We mainly used an empty needle to aspirate approximately 100ml of cerebrospinal fluid every day to reduce intracranial pressure. The formation of LPH in this patient might be due to 30 sessions of craniocerebral radiotherapy. Standard 60 Gy in 30 fractions is widely used and not uniquely associated with LPH, though cranial RT can contribute to leukoencephalopathy and hydrocephalus. There are many contradictions in the treatment of this type of patients. As patients after glioma surgery need radiotherapy and chemotherapy, they gradually develop complications such as bone marrow suppression and hydrocephalus. With the recurrence of the tumor, these symptoms gradually worsen, eventually leading to a very poor prognosis for the patients. In our study, we engaged in comprehensive discussions and analyses regarding the etiologies of LPH in the patients, which are detailed as follows: 1. Compliance Hypothesis: It is posited that high-dose focal radiotherapy (60 Gy in 30 fractions) may induce diffuse microvascular injury and astrocytic dysfunction, consequently reducing the brain’s elastic modulus. A “softer” brain necessitates a lower ICP to maintain ventricular patency, resulting in ventricular dilation despite ICP measurements remaining below 5 cmH_2_O. 2. Cerebrospinal Fluid (CSF) Leak Hypothesis: The surgical cavity located in the left temporal pole was directly continuous with the subarachnoid space and required only secondary sealing due to extensive dural invasion, precluding a watertight closure. We hypothesize the presence of a persistent “micro-fistula” between the ventricular system and the extracranial subgaleal space, functioning as a low-resistance outlet and maintaining the system in a chronic state of under-pressure. This hypothesis accounts for the ease of CSF aspiration from the external drain while spontaneous flow is absent. 3. Choroid Plexus Hyposecretion: Previous research has demonstrated that single-fraction doses of ≥8 Gy can reduce choroid plexus Na^+^/K^+^-ATPase activity by 30–40%, resulting in a 15–25% decrease in daily CSF production. Although our patient received fractionated radiotherapy, the cumulative dose exceeded the threshold reported for secretory decline, possibly adding a “CSF hypoproduction” component to the above mechanisms. The decision to perform a lateral ventricle drilling and drainage procedure, followed by repeated CSF drainage, was necessitated by the patient’s sudden cardiac arrest prior to surgery and the subsequent CT scan, which indicated a worsening of hydrocephalus. To temporarily mitigate the increased intracranial pressure, drilling and drainage were employed as an alternative to shunt surgery. However, post-procedure, the CSF did not flow as expected through the drainage tube. Consequently, daily aspiration of CSF using an empty needle was undertaken to alleviate symptoms of hydrocephalus and increased intracranial pressure, thereby preventing cerebral herniation. Previous studies have documented that patients experiencing acute LPH often progress to chronic, typical non-absorbable hydrocephalus, at which point a ventriculoperitoneal shunt is deemed appropriate (19). If the patient progresses from the acute phase of recurrent cerebrospinal fluid aspiration-induced LPH to the chronic stage, we may further perform a conventional shunt surgery to treat the patient. We also systematically summarized the differential diagnoses for the patient’s enlarged ventricles in this case (**Supplementary Table 1**).

This case report describes three unusual features: (1) incidental diagnosis of GBM two months after surgical treatment for TBI, (2) development of suspected low-pressure hydrocephalus (LPH) following standard chemoradiotherapy, and (3) fatal outcome due to diffuse cerebral edema and brain herniation despite aggressive intervention. To our knowledge, this case appears to be uncommon combining these elements.

In addition, this report has several important limitations. First, it is a single retrospective case report without generalizability. Second, no pre-TBI brain imaging was available, making it impossible to determine whether the GBM was pre-existing. Third, ICP was not measured, so the diagnosis of LPH remains suspected rather than confirmed. Fourth, no autopsy was performed to confirm the cause of death or evaluate the hypothesized CSF micro-leak. Fifth, bone marrow suppression was attributed to chemotherapy without confirmatory bone marrow biopsy. At last, the patient perspective could not be obtained as the patient died prior to the completion of this report.

Although our report does not provide epidemiological data and lacks generalizability, documenting the occurrence, progression, clinical features, complications, and prognosis of such GBM cases may offer valuable guidance for future patient management.

## Conclusion

In conclusion, this report is the first to document a case in which a glioblastoma was identified two months following a brain trauma, ultimately resulting in patient mortality despite extensive therapeutic interventions due to tumor recurrence, bone marrow suppression, and suspected low-pressure hydrocephalus. This case provides a reference point for the incidental discovery and subsequent management of asymptomatic GBM, while also offering insights into treatment strategies and research directions for suspected low-pressure hydrocephalus that emerges during disease progression. There is an urgent need for further prospective studies and case reports to elucidate the epidemiology, pathogenesis, diagnosis, and treatment of this severe GBM complication. Moreover, additional research is necessary to explore the potential correlations and mechanisms linking TBI with the onset and progression of GBM.

## Data Availability

The datasets presented in this article are not readily available because of ethical and privacy restrictions. Requests to access the datasets should be directed to the corresponding author/s.
