# Supplementary material for: Case Report: Suspected low-pressure hydrocephalus following surgical treatment of traumatic brain injury in a patient with subsequently diagnosed glioblastoma
Source: Front Oncol. 2026 Jun 29;16:1721506. doi: 10.3389/fonc.2026.1721506 (PMC13357208; doi:10.3389/fonc.2026.1721506)
Supplement: Supplementary file 1 [file DataSheet1.docx]

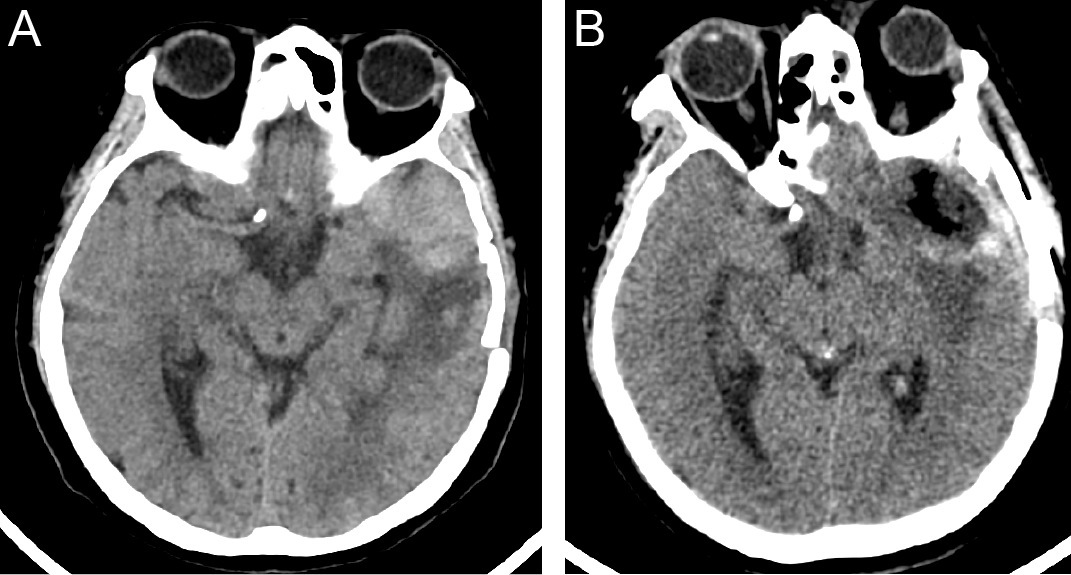


Figure S1. Head CT before and after the first operation.


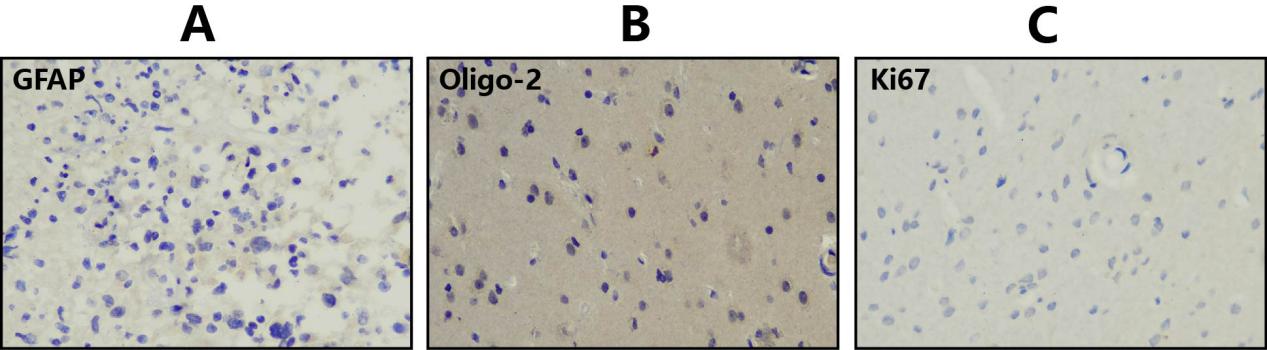


Figure S2. Immunohistochemistry for the indicated proteins of the patient. A: GFAP; B: Oligo-2; C:Ki67.


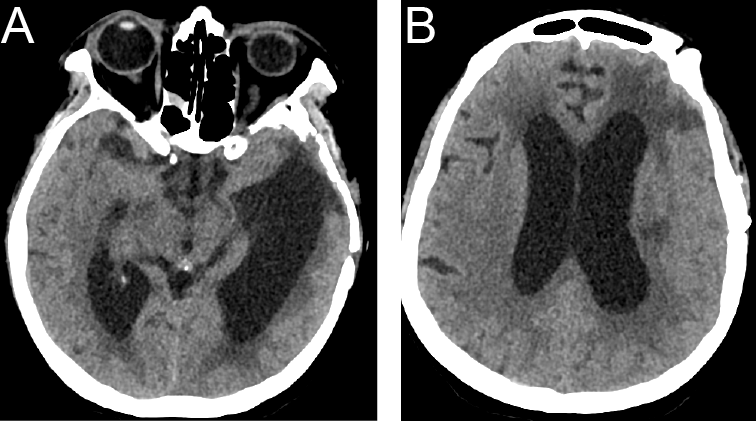


Figure S3. Head CT half a year after the first surgery.

| Diagnostic possibility | Supporting evidence | Evidence against | How it was (or could have been) evaluated |
| --- | --- | --- | --- |
| Low-pressure hydrocephalus (LPH) | Poor spontaneous EVD drainage; ease of manual aspiration; ventriculomegaly out of proportion to clinical status | No direct ICP measurement; no ICP <5 cmH₂O documented | ICP monitoring (not performed); response to negative-pressure drainage |
| Normal-pressure hydrocephalus (NPH) | Ventriculomegaly; possible gait/cognitive disturbance | Acute/subacute course; post-radiotherapy context | ICP monitoring; CSF tap test (not performed) |
| High-pressure hydrocephalus | History of GBM; tumor recurrence; post-hemorrhagic etiology | Poor spontaneous EVD drainage (would be brisk in high pressure) | ICP monitoring (not performed); EVD opening pressure (not recorded) |
| Hydrocephalus ex vacuo | Diffuse brain swelling on final CT | Ventricular enlargement preceded diffuse edema; not purely compensatory | Serial imaging review |
| CSF circulation disturbance post-radiotherapy | Known complication of cranial RT (60 Gy) | Typically presents as NPH, not acute LPH | CSF flow studies (not performed) |
| CSF micro-leak | Dural repair not watertight; surgical cavity continuous with subarachnoid space | No overt CSF rhinorrhea or wound leak | Radioisotope cisternography (not performed) |
| Post-resuscitation changes | Cardiac arrest with hypoxia preceding worsening hydrocephalus | Ventricular enlargement began at 4 months (before cardiac arrest at 8 months) | Temporal analysis |
| Tumor recurrence obstructing CSF pathways | MRI at 4 months showed minor recurrence; CT at 8 months showed significant recurrence | Ventricular enlargement preceded major recurrence | Serial imaging; CSF flow studies |

Table S1. Differential diagnosis of ventricular enlargement in the present case.
